# Pro-Viral and Anti-Viral Roles of the RNA-Binding Protein G3BP1

**DOI:** 10.3390/v15020449

**Published:** 2023-02-06

**Authors:** Aravinth Kumar Jayabalan, Diane E. Griffin, Anthony K. L. Leung

**Affiliations:** 1 Department of Biochemistry and Molecular Biology, Bloomberg School of Public Health, Johns Hopkins University, Baltimore, MD 21205, USA; 2 W. Harry Feinstone Department of Molecular Microbiology and Immunology, Bloomberg School of Public Health, Johns Hopkins University, Baltimore, MD 21205, USA; 3 McKusick-Nathans Department of Genetics Medicine, School of Medicine, Johns Hopkins University, Baltimore, MD 21205, USA; 4 Department of Oncology, School of Medicine, Johns Hopkins University, Baltimore, MD 21205, USA; 5 Department of Molecular Biology and Genetics, School of Medicine, Johns Hopkins University, Baltimore, MD 21205, USA

**Keywords:** G3BP1, stress granules, innate immune response, proviral, antiviral, condensates, RNA-binding protein

## Abstract

Viruses depend on host cellular resources to replicate. Interaction between viral and host proteins is essential for the pathogens to ward off immune responses as well as for virus propagation within the infected cells. While different viruses employ unique strategies to interact with diverse sets of host proteins, the multifunctional RNA-binding protein G3BP1 is one of the common targets for many viruses. G3BP1 controls several key cellular processes, including mRNA stability, translation, and immune responses. G3BP1 also serves as the central hub for the protein–protein and protein–RNA interactions within a class of biomolecular condensates called stress granules (SGs) during stress conditions, including viral infection. Increasing evidence suggests that viruses utilize distinct strategies to modulate G3BP1 function—either by degradation, sequestration, or redistribution—and control the viral life cycle positively and negatively. In this review, we summarize the pro-viral and anti-viral roles of G3BP1 during infection among different viral families.

## 1. Introduction

Eukaryotic cells reprogram a variety of cellular processes and pathways in response to unfavorable growth conditions, including viral infection. When stressed, cells selectively transcribe and translate stress-responsive genes while arresting global protein synthesis to conserve energy [1,2,3,4]. Upon viral entry, cells trigger innate response pathways to eliminate or prevent viral replication [5]. The first line of host defense against viral infection is the recognition of viral RNA or DNA by pathogen-associated molecular patterns (PAMPs) through different sensors [6]. For example, viral nucleic acids can be detected by retinoic acid-inducible gene I (RIG-I)-like receptors (RLRs), Toll-like receptors (TLRs), and cGMP/cAMP synthase (cGAS) [7,8,9,10,11,12]. These proteins detect and distinguish viral vs. cellular nucleic acids based on the structural differences, such as the presence of a 5′-triphosphate (5′-PPP) moiety in viral RNAs [13]. In turn, viruses have developed adaptive mechanisms to counteract host innate responses for successful propagation [14]. Even the most complex virus encodes only a few hundred proteins; thus, viruses must interact with host proteins to exploit their cellular machinery for efficient viral replication [14,15,16].

Among the host proteins, ribosomes and translation factors are critical for viral protein synthesis and mRNA binding proteins to stabilize viral RNA, and some proteins are involved in the viral replication complexes. The Ras GTPase-activating (SH3 domain) protein-binding protein G3BP1 is an RNA-binding protein targeted by many viruses [17]. G3BP1 is highly conserved from yeast to humans and plays a key role in RNA metabolism, such as regulating the stability and translation of a subset of mRNAs, binding to specific transcripts during stress conditions, and activating interferon (IFN)-stimulated gene expression [18,19,20,21]. G3BP1 function is further modulated by post-translational modifications and also implicated in many diseases such as cancer, neurodegeneration, and viral infection—the latter is the focus of this review [22,23,24,25,26].

**Figure 1:**
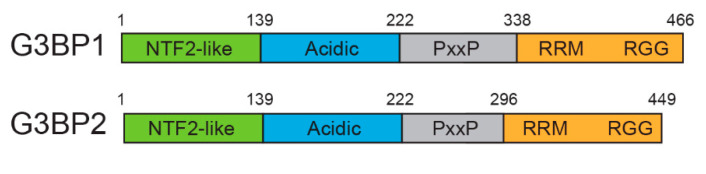
Domain architecture of G3BP1 and G3BP2.

G3BP1 and its paralog G3BP2 possess similar protein domain architecture composed of regions for protein, RNA and DNA interactions (Figure 1). These domains include N-terminal nuclear transport factor 2-like (NTF2L), followed by an acidic domain, proline-rich region (PxxP), an RNA recognition motif (RRM), and an arginine and glycine-rich region (RGG) at the C-terminus [27,28]. The N-terminal domains contain motifs for protein binding while the C-terminal domains bind nucleic acids. For instance, the NTF2L domain interacts with FGDF sequence motifs present in many proteins and the PxxP domain interacts with SH3 motifs. The RRM domain is essential for RNA binding and RGG domain increases RNA binding as well as mediates protein–protein interactions. The functions of G3BP1 and G3BP2 are redundant in some, but not all cases [29]. For example, knockdown of G3BP1 increases the expression of G3BP2 to compensate for the loss of G3BP1 expression and function, but not vice versa [23,29].

Emergent data suggest that G3BP1 is one of the key proteins that impact the viral life cycle both positively and negatively [30,31]. For example, some viruses require G3BP1 for efficient viral replication, while the replication of other viruses is negatively affected by G3BP1 expression. Well-studied functions of G3BP1 include participation in the innate response to infection through activation of viral nucleic acid sensors RIG-I and cGAS and as an essential component of stress granules (SGs) [29,30,32,33,34,35]. SGs are a class of cytoplasmic biomolecular condensates enriched with mRNA–protein complexes assembled in response to stress, including viral infection. Knockout of both G3BP1 and G3BP2 is necessary to completely inhibit SG formation induced by many stressors, implying their importance to form protein–protein and protein–RNA interactions within SGs [29]. Given that G3BP1 impacts the life cycle of many viruses, it is important to distinguish what specific functional roles are played by G3BP1 at different infection stages. Here, we focus on the role of G3BP1 during viral infection and the various strategies utilized by viruses to exploit or counteract G3BP1 functions.

## 2. G3BP1 Amplifies Innate Immune Signaling

**Figure 2:**
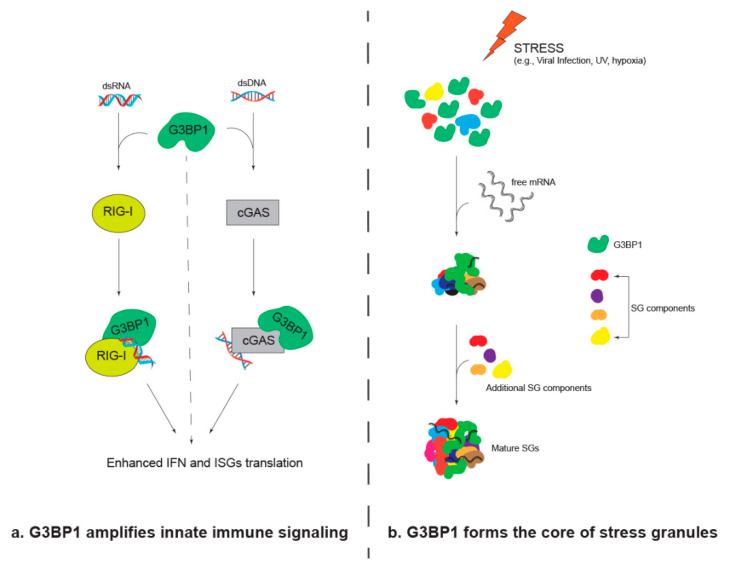
Role of G3BP1 in (**a**) innate immune signaling and (**b**) stress granule assembly.

G3BP1 regulates early innate responses through its ability to bind nucleic acids and proteins in these signaling pathways. G3BP1 facilitates the initial IFN-β response by binding and augmenting the function of viral RNA and DNA sensing proteins in the cytoplasm. For example, the RGG domain of G3BP1 binds the RIG-I helicase domain and viral dsRNA to prevent RIG-I degradation and enhance *Ifn-b* mRNA expression [30]. G3BP1 also interacts with cGAS to form large complexes that enhance interaction with viral DNA to induce IFN [30,34,36]. The binding of the N-terminal region of cGAS with G3BP1 is required for activation of the RNA-dependent protein kinase PKR leading to co-localization as small cytoplasmic foci [35]. The local concentration of G3BP1, cGAS, and PKR within cytoplasmic foci is proposed to enhance PKR activity. G3BP1 is required for activation of cGAS as depletion of G3BP1 dramatically reduces DNA-induced IFN production [34]. These data suggest that G3BP1-mediated interactions with signaling molecules facilitate the initiation of downstream innate response pathway signaling. G3BP1 can also facilitate the expression and translation of IFN-stimulated genes (ISGs). For example, G3BP1, G3BP2, and their binding partner Caprin1 positively regulate the translation of multiple ISGs in response to IFN (Figure 2a) [21].

## 3. G3BP1 Forms the Core of Stress Granules

A well-known function of G3BP1 during stress stimuli is its involvement in SG assembly (Figure 2b). SGs assemble upon stress-induced translation arrest that results in the accumulation of free untranslated mRNAs and mRNA binding proteins in the cytoplasm [37]. Upon stress, one of the following five kinases is activated: double-stranded RNA (dsRNA)-dependent PKR, protein kinase R-like endoplasmic reticulum kinase (PERK), heme-regulated inhibitor (HRI), general control nonderepressible-2 (GCN2), or microtubule affinity-regulating kinase 2 (MARK2). The activated kinase phosphorylates the translation initiation factor eIF2α subunit [38,39]. The initiation complex containing the phosphorylated eIF2α becomes unavailable for recycling, which leads to a stall in translation initiation [40]. Once the elongating ribosomes run off from polysomes, the stalled translation initiation results in a sudden influx of untranslated mRNAs in the cytoplasm. These untranslated mRNAs bound with ribonucleoproteins (mRNPs) cluster together with G3BP1, G3BP2, and several other RNA binding proteins to form RNA/protein-rich condensates in the cytoplasm. Several post-translational modifications (PTMs) control the dynamics of SGs [33,41,42,43,44,45,46]. Of note, G3BP1 itself undergoes several post-translational modifications, including phosphorylation, methylation, ubiquitination, and ADP-ribosylation during stress conditions [42,44,47]. G3BP1 and G3BP2 form the core protein–protein interaction network which binds translation factors, mRNA binding proteins, long and translationally incompetent mRNAs as well as specific transcripts during stress conditions [19,48,49]. RNAi-mediated knockdown or genetic knockout of both G3BP1 and G3BP2 results in cells that do not form SGs in response to most stressors, emphasizing the importance of G3BP1/G3BP2 in SG assembly [29]. These data suggest that G3BP1/G3BP2 act as a scaffold that recruits other proteins into SGs [47,50]. 

Recent studies provided more mechanistic insights into the role of G3BP1 as the scaffold for SG assembly. G3BP1 is the central node of the protein–protein interaction network within SGs, where RNA serves as a molecular trigger for phase separation. In the normal state, G3BP1 adopts a compact, closed auto-inhibitory state with intramolecular interaction between acidic and arginine-rich regions. Increasing local RNA concentrations can increase their binding to G3BP1, relieving G3BP1 from the autoinhibitory state and resulting in more RNA recruitment and less aberrant RNA–RNA interaction [50,51,52]. In light of the viral infection, it may be possible that at the early infection stage, an increase in viral RNA concentration may relieve G3BP1 from its auto-inhibitory state, triggering G3BP1 condensation, with the protein serving as the scaffold to recruit other proteins/RNA required for viral replication.

## 4. Role of G3BP1 during Viral Infection

**Figure 3:**
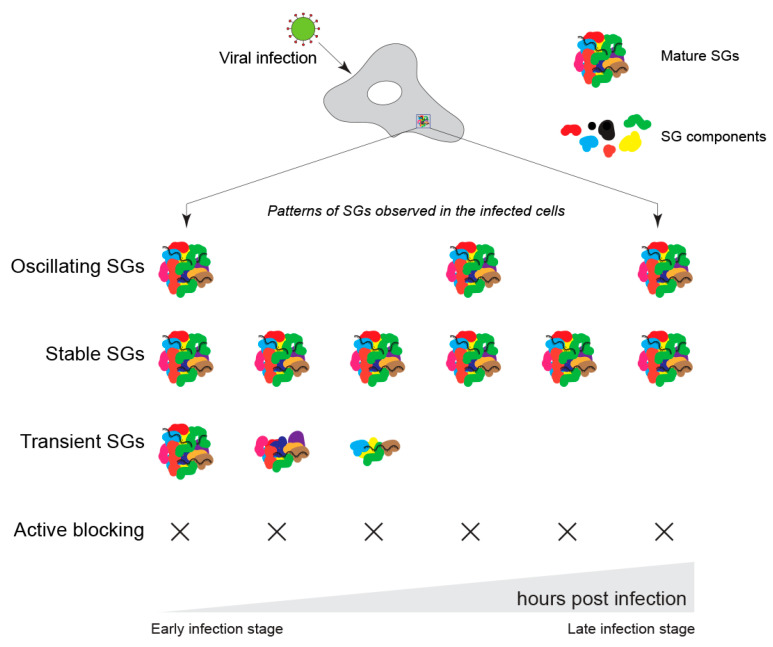
Patterns of SGs observed during infection by distinct viral types.

At least four distinct temporal patterns of SGs have been observed during infection with different viruses: (1) stable, (2) transient, (3) oscillating, or (4) no SGs (active blocking of SG induction) [53,54,55]. These patterns highlight the importance of modulating SGs upon virus infection (Table 1). As demonstrated by one of the earliest studies using Semliki Forest virus (SFV), infection-induced translation arrest results in the assembly of transient SGs. Given that some of the key anti-viral proteins (such as ADAR1, PARP13/ZAP, OAS, IRF3, IRF7, TBK1, and RNase L) localize to SGs and activate the anti-viral response, SGs may negatively affect viral replication [56,57,58,59,60]. Thus, viruses may target SGs not only to modulate G3BP1 function but also to disassemble SGs to counteract anti-viral mechanisms in the infected cells. On the other hand, the presence of SGs may facilitate the switch from viral translation to genome amplification by sequestering translation factors into the condensed state (Figure 3) [23].

**Figure 4:**
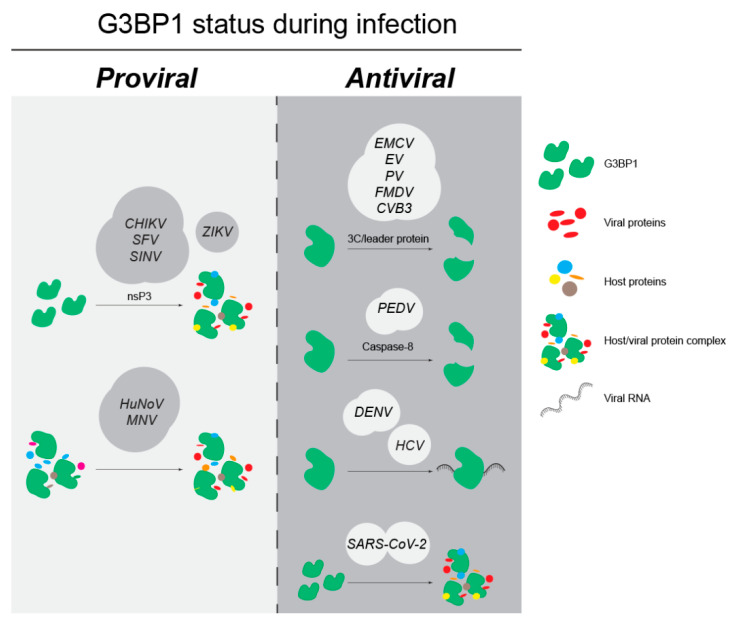
Strategies employed by different viruses to modulate G3BP1.

G3BP1 can either promote or inhibit the viral life cycle, but the molecular mechanisms are not completely understood. One way by which G3BP1 may regulate its function during infection is through post-translational modifications. For example, ADP-ribosylated G3BP1 is crucial for SG assembly, given that *chikungunya virus* (CHIKV) actively reduces G3BP1 ADP-ribosylation, disrupting SGs with translation factors released from the condensed state [47]. However, G3BP1 is also required for establishing CHIKV replication complexes, and post-translational modifications required for this process have not been defined [23]. Thus, post-translationally modified G3BP1 may act as an anti-viral protein, while the unmodified or differently modified G3BP1 could play a pro-viral role during CHIKV infection. Similarly, arginine methylation status of G3BP1 partly recruits Tudor domain-containing protein (TDRD3) to regulate innate immune response against *enterovirus* species [60]. Using polyinosinic:polycytidylic acid as a mimic for viral replication double-stranded intermediates, Kim et al. showed that G3BP1 is phosphorylated at tyrosine residue 40 by Bruton’s tyrosine kinase (BTK). Phosphorylation of G3BP1 by BTK is critical for dimerization and phase separation to limit viral spread [88]. Further clues are obtained from identifying the strategies that viruses employ to target G3BP1—i.e., sequestration, cleavage, or degradation—and modulate its function during infection (Figure 4). In general, viruses use their genome-encoded proteins to sequester G3BP1 if it is required for viral replication. On the contrary, viruses cleave G3BP1 when it exerts an anti-viral role (Table 2). Here, we describe the role of G3BP1 during specific viral infections and the ways by which G3BP1 is targeted.

G3BP1 controls several key cellular processes, including mRNA metabolism, ribosomal quality control, immune response, and SG assembly. G3BP1 facilitates replication of some viruses whereas it restricts viral spread in other viruses. Depending on the virus type, G3BP1 plays different roles in the viral life cycle, including (1) stabilizing viral RNA; (2) recruiting ribosomes to initiate viral RNA translation; (3) serving as a scaffold to build viral replication complexes; (4) assembling SGs; (5) stabilizing and enhancing the IFN-β response; and (6) activating cGAS and RIG-I pathways. Here, we explore the mechanisms by which different viruses regulate G3BP1 function during infection.

### 4.1. G3BP1 as an Anti-Viral Factor

G3BP1 enhances the immune response against invading viruses by inducing the expression of IFN and translation of ISGs. In virus-infected cells, G3BP1 binds to dsRNA resulting from the viral replication intermediates through the RGG domain and elevates RIG-I induced IFN-ꞵ mRNA expression [30]. In response to IFN, G3BP1, G3BP2, and Caprin 1 promote ISG translation to synthesize anti-viral factors. G3BP1 also assembles SGs in response to infection-mediated translation arrest and limits the availability of translation factors for viral protein synthesis. To counter these anti-viral functions, viruses target G3BP1—either cleaving or sequestering it during infection. Here we summarize the strategies shared by viruses in the *Picornaviridae* and *Coronaviridae* families to counteract host defense. 

#### 4.1.1. Picornaviruses Inhibit G3BP1 Function through Protease-Mediated Cleavage

*Picornaviruses* are small, non-enveloped, positive-sense RNA viruses that include EMCV, EV, PV, FMDV, and CVB3. G3BP1 negatively regulates *Picornaviridae* infection by binding the 3′ untranslated regions (3′UTRs) or Internal Ribosome Entry Site (IRES) regions of viral RNAs to inhibit viral replication [53,95]. To counteract these effects, the 3C protease or leader protein of these viruses cleaves G3BP1 between Q325 and Q326 at late infection stages. G3BP1 cleavage is essential for the expression of viral proteins, and expression of the non-cleavable G3BP1 Q325E mutant suppresses viral replication [61,62,63,64,65,66,67,68,69]. 

During *Picornaviruses* infection, PKR is activated by double-stranded RNA intermediates, phosphorylates eIF2α, and induces SG formation. SGs are observed early in infection but disassembled at late stages of infection. SG disassembly correlates temporally with G3BP1 cleavage, and the expression of the non-cleavable G3BP1 Q325E mutant results in sustained IFN-ꞵ mRNA expression and SG persistence. Consistent with the observed effect on G3BP1 cleavage, knockdown of G3BP1 increases viral protein synthesis and virus production, whereas G3BP1 overexpression shows an inhibitory effect [61,64,65,66]. 

FMDV, which uses the leader protein to cleave G3BP1, also causes SG disassembly. Catalytic mutant C41A or leaderless FMDV inefficiently inhibits arsenite-induced SG formation in infected cells [63]. FMDV infection also causes degradation of G3BP1 through autophagy [62]. Thus, protease-mediated cleavage or degradation of G3BP1 is the fundamental mechanism employed by *Picornaviridae* to restrict anti-viral activities of G3BP1. 

#### 4.1.2. Coronaviruses Modulate G3BP1 Function Either by Cleavage or Sequestration 

*Coronaviridae* is a family of enveloped viruses with large single-stranded positive-sense RNA genomes and includes PEDV and SARS-CoV-2. Similar to *Picornaviridae*, G3BP1 negatively regulates *coronavirus* infection. Overexpression of G3BP1 reduces viral replication, protein synthesis, and virus production, while G3BP1 knockdown enhances viral replication. However, these viruses utilize different strategies to modulate G3BP1 function; for example, PEDV induces cleavage of G3BP1 between D168 and D169 by caspase-8 to regulate SG dynamics in infected cells. Expression of non-cleavable G3BP1 results in persistent SGs and reduces viral replication, but the mechanism behind G3BP1-mediated reduction of viral replication remains unexplored [79,80].

SARS-CoV-2—the virus responsible for the COVID-19 pandemic—modulates G3BP1 function by sequestration and condensation with the nucleocapsid protein. The intrinsically disordered region (IDR) at the N-terminal region of nucleocapsid is required for condensation with G3BP1, and this IDR is also crucial for viral particle production. Though it is unclear whether SARS-CoV-2 induces SGs, G3BP1–nucleocapsid binding blocks the interaction of SG proteins such as PKR and USP10 with G3BP1 in arsenite-treated cells [82]. However, the role of G3BP1 remains unclear given the conflicting reports on the effect of G3BP1 knockdown in structural protein and viral RNA levels [81,82,83,96]. Recent studies highlighted the potential anti-viral role of G3BP1 in the lung tissue of COVID-19 patients and SARS-CoV-2-infected mice. In COVID-19 patients and mice, the N protein interacts with G3BP1, suppresses SG formation, and potentiates viral infection by antagonizing the G3BP1-mediated host innate immune response pathway [97,98].

### 4.2. G3BP1 as a Pro-Viral Factor

Although G3BP1 synergizes host immune responses to fight viral invasion, some viruses require G3BP1 for efficient infection. Such pro-viral activities have been reported for several viruses including CHIKV, *hepatitis C virus*, and *respiratory syncytial virus* [23,74,99]. For example, during CHIKV infection, depletion of both G3BP1 and G3BP2 significantly decreases viral RNA levels, protein expression, and subsequent viral progeny production [23]. Binding of the viral genome by G3BP1 and sequestering it into viral replication complexes may be important to prevent G3BP1-mediated activation of the innate immune response. The proposed pro-viral functions of G3BP1 include: stabilizing viral mRNAs to prevent degradation, facilitating viral protein translation by recruiting translation factors and ribosomes, acting as a scaffold to build viral replication complexes, and amplifying the viral genome. In this section, we will discuss the different strategies employed by viruses that use G3BP1 to promote viral replication. 

#### 4.2.1. Togaviruses Sequester G3BP1 to Facilitate Viral Replication and Translation

*Togaviridae* is a family of enveloped, icosahedral viruses with a positive-sense single-stranded RNA genome including the mosquito-borne alphaviruses that cause rash and arthritis such as CHIKV, SFV, and SINV. Notably, the alphavirus genus can be categorized into the Old World alphaviruses (CHIKV, SINV, SFV) and the New World alphaviruses (VEEV, EEEV) based on their geographical origin. Cells lacking both G3BP1/G3BP2 do not support replication of the Old World alphaviruses but do support replication of the New World encephalitic alphaviruses, indicating a virus-specific importance of G3BP1 for infection. In infected cells, G3BP1 is sequestered through interaction of the NTF2L domain with FGDF motifs in the C-terminal hypervariable region of non-structural protein 3 (nsP3). For initiation of infection, G3BP1 facilitates CHIKV, but not to SFV, translation of genomic RNA by enriching translation factors at cytopathic vacuoles [100]. The binding of nsP3 with G3BP1 is a proposed mechanism for SG disassembly where nsP3-G3BP1 binding inhibits the interaction between other SG components and G3BP1. However, SFV without FGDF motifs still disassembles SGs, but at a slower rate suggesting an additional mechanism for SG disassembly [77]. Recently, our group discovered that the SG disassembly for CHIKV is partly mediated by the macrodomain at the N-terminus of nsP3. The macrodomain possesses ADP-ribosylhydrolase activity (the ability to remove ADP-ribose from conjugated proteins), and this enzymatic activity is critical for replication and virulence of viruses from several viral families [92,101,102,103,104]. G3BP1 ADP-ribosylation, a key driver of SG assembly, is reduced in cells infected by wild-type CHIKV, but not a mutant virus that lacks hydrolase activity. Infection with the mutant virus results in delayed SG disassembly and reduced viral structural protein synthesis in neuronal cells, suggesting that G3BP1 ADP-ribosylation regulates virus production [47,105]. 

Notably, the New World alphaviruses do not have the FGDF motif and thus do not bind G3BP1. Instead, these viruses sequester other SG components, such as FXR1, FXR2, and FMR1, which bind to ribosomes. Hence, these proteins possibly recruit ribosomes and facilitate translation of New World viral genomic RNA similar to the G3BP1 role for Old World alphaviruses [78,106]. Therefore, although SG components are targeted by both Old World and New World alphaviruses, G3BP1 plays a pro-viral role only for Old World alphaviruses.

#### 4.2.2. Caliciviruses Remodel the G3BP1 Interactome during Infection

The *Caliciviridae* family comprises small non-enveloped positive-strand RNA viruses, including *human norovirus* (HuNoV) and the closely related *murine norovirus* (MNV). MNV remodels G3BP1 interactome in infected cells and induces cytoplasmic G3BP1 granules during infection that are distinct from SGs. The proteome of G3BP1 granules contains several proteins that are present in viral replication complexes, suggesting a pro-viral role of G3BP1 during MNV infection. Consistently, genetic depletion of G3BP1 severely affects viral replication and translation. In addition, knockdown of MNV-induced G3BP1 interactors reduces MNV replication, suggesting that the G3BP1 interactome consists of host factors that are required for efficient viral replication complex formation [84,107]. Similar to alphaviruses, G3BP1 in MNV-infected cells recruit ribosomes to viral RNA and initiates translation [31]. During MNV infection, both PKR and GCN2 phosphorylate eIF2α; however, SG formation is not observed in the infected cells, even though they can still form SGs upon arsenite treatment, suggesting that MNV decouples the SG formation from eIF2α phosphorylation during virus infection. In contrast, *feline calicivirus* cleaves G3BP1 through the viral 3C-like proteinase NS6^Pro^ [94].

### 4.3. G3BP1 Is Differentially Utilized within the Flaviviridae Family

Although G3BP1 influences viral propagation of *Flaviviridae* family, different members utilize G3BP1 at different stages of infection. In this section, we describe how G3BP1 function is modulated by DENV, ZIKV, and HCV.

#### 4.3.1. Dengue Virus (DENV) Modulates G3BP1 Function by Interacting with 3′UTR vRNA

DENV infection induces G3BP1 punctate-like structures at the early stage of infection. Although the phosphorylation of eIF2α by PKR occurs at the late infection stage [73], it is unclear whether infected cells induce SGs. The expression of viral protein is reduced in cells containing G3BP1 granules, whereas knockdown of G3BP1 enhances protein level and viral titers. In DENV-2-infected cells, G3BP1 is also associated with ISG mRNAs and facilitates their translation to inhibit viral replication. As an IFN countermeasure during DENV-2 infection of Huh-7 cells, a long noncoding RNA from the subgenomic region of flaviviral RNA (sfRNA) produced by all flaviviruses binds G3BP1, G3BP2, and Caprin1, and thereby inhibits the translation of ISGs [21]. Not all flavivirus sfRNAs have this property and whether a similar decoy phenomenon may be used by other viruses to counter the production of ISG proteins and promote viral replication is unknown.

#### 4.3.2. ZIKV and HCV Require G3BP1 for Proper Viral Replication

In both ZIKV- and HCV-infected cells, G3BP1 associates with the viral replication complex. G3BP1 depletion reduces viral replication, titer, and viral protein levels, while overexpression of G3BP1 enhances viral titers. In addition, in ZIKV-infected cells, G3BP1 interacts with capsid protein and co-localizes with envelope protein, suggesting a possible role for G3BP1 in virion assembly [70,71,72]. During HCV infection, G3BP1 interacts with the 5′-UTR of viral RNA and is involved in genome amplification [74]. Interestingly, HCV requires only G3BP1, but not G3BP2 whose knockdown does not alter viral replication [74,75].

### 4.4. Viruses without a Defined Role for G3BP1 in Infection

Though G3BP1 is a predominant target for many viruses, there are some viruses whose replication is unaffected by the expression level of G3BP1. 

#### 4.4.1. Depletion or Overexpression of G3BP1 Does Not Affect PRRSV Infection

PRRSV—a single-stranded positive-sense enveloped virus in the *Arteriviridae* family—does not require G3BP1, yet the protein is closely associated with viral replication complexes. Neither deletion nor overexpression of G3BP1, G3BP2, or both affects viral titers. In contrast to the above-described viruses, PRRSV induces SGs only at late stages of infection (48–72-h post-infection) through PERK-, rather than PKR-mediated eIF2α phosphorylation. The reason for G3BP1 association with viral replication complexes and the mechanism and role of SG assembly at late infection stages are not known [85,86]. Intriguingly, one study suggested a pro-viral function of SG assembly during PRRSV infection. Cells infected with PRRSV induces SGs, recruits G3BP1 and the viral replicase protein nsP1β, thereby inhibiting G3BP1-mediated PKR activation [108].

#### 4.4.2. G3BP1 Is Sequestered within Ebola Virus Inclusions

EBOV, a single-stranded negative-sense RNA virus, does not induce SG assembly but sequesters SG components G3BP1, eEF2, eIF2, and eIF3 through the viral protein VP5 **[55,87]**. Although infection-mediated SG assembly is not observed, EBOV inhibits arsenite-induced SG assembly. VP5 expression affects IFNɑ/ꞵ expression and PKR kinase activation in infected cells. Although G3BP1 is sequestered within the viral inclusions, the exact role of G3BP1 in EBOV infection is not well understood.

## 5. Discussion

Viruses employ at least three strategies to target and modulate G3BP1 function during infection: (1) enzymatic activity such as proteolytic cleavage or removing post-translation modification (e.g., poliovirus and alphavirus); (2) binding or sequestration (e.g., alphavirus and coronavirus); and (3) redistribution or remodeling of G3BP1 interaction (e.g., norovirus). These functional modulations of G3BP1 by viruses impacts the viral life cycle either positively or negatively. In general, if G3BP1 exerts anti-viral activity, then viruses cleave or sequester G3BP1 (e.g., polioviruses, flaviviruses). If G3BP1 is required for viral replication, viruses do not cleave G3BP1, but instead recruit it to build viral replication complexes (e.g., alphaviruses). For pro-viral roles, the NTF2L and RGG domains of G3BP1 play a crucial role to bind viral proteins or viral RNA, respectively. Besides, some viruses possess conserved motifs (e.g., FGDF) within their proteins to bind and modulate G3BP1 function [76,109]. Intriguingly, the post-translational modification status of heavily regulated G3BP1 may determine the functional consequences of interaction. G3BP1 methylation recruits TDRD3 into SGs to regulate innate immune response [60]. Alphaviruses and coronaviruses encode an enzyme to remove ADP-ribosylation indicating that the modified form of G3BP1 may possess anti-viral functions. Given that G3BP1 is also modified by phosphorylation and ubiquitylation, it is worth checking the status of G3BP1 for other post-translation modification at different infection stages and determine how these modifications affect a viral life cycle [33,42]. Furthermore, the differences in the role of G3BP1 during infection—pro-viral or anti-viral—may also depend on the type and differentiation status of the cell infected.

G3BP1 plays critical yet distinct roles during infection by different families of the virus. Recent proteomic studies provided some insights into the G3BP1 interactome during infection. In SINV-infected cells, G3BP1 stably interacts with viral proteins throughout the life cycle, whereas some SG components interact in a time-dependent manner. During norovirus infection, the G3BP1 interactome is modulated to inhibit the formation of SGs. Given that the interacting partners of G3BP1 are modulated during infection to form new complexes in a virus-specific manner, different terms have been used to differentiate for these viral-induced protein complexes, including atypical SGs (EV), anti-viral SGs (IBV), or nsP3 foci (alphaviruses). Therefore, not all G3BP1-positive structures are SGs, and should be stained with other markers to ascertain their identities. Unlike RNA viruses, the role of G3BP1 during infection with DNA viruses is poorly understood. For example, in *vaccinia virus*-infected cells, G3BP1 and other SG components are recruited to cytoplasmic viral factories where transcription and translation occur. However, the precise function of G3BP1 in these viral factories is yet to be characterized. Extensive research is warranted to delineate whether G3BP1 plays a pro-viral or anti-viral role in DNA viruses [110,111].

Finally, it is noteworthy to mention that some studies have been performed in a system by overexpressing or knocking down G3BP1, which may result in ambiguous conclusions. For example, G3BP1 knockdown increases G3BP2 expression, while G3BP1 overexpression induces PKR-mediated eIF2α phosphorylation and SG formation–these non-physiological changes could affect the normal stages of the viral life cycle. To overcome these concerns, many groups recently have begun to apply CRISPR technologies to genetically knock in a fluorescent tag or knock out G3BP1 to delineate the functions of G3BP1 under physiological conditions. Special consideration should also be given to the cell type and viral load, as these factors may influence the outcome of virus infection. Given that these factors also modulate the cellular status and proteome in a spatio-temporal manner, it is critical to analyze at single-cell levels using live-cell imaging, and assays, such as ribopuromycylation, to quantitate the cellular changes only in the infected cells. Given that G3BP1 is a well-described target for many viruses, clarifying the precise functional roles of G3BP1 during infection will not only advance the field of host-virus interaction but may also offer novel therapeutic opportunities.

## Figures and Tables

**Table 1:** SG status during infection against different viruses.

Family	Species	SG Dynamics	Duration Monitored for SG Presence	eIF2alphα Phosphorylation Status	Responsible Kinase	Cell Line Tested	References
Picornaviridae	EMCV	Transient	4, 12 hpi	*ND*	PKR	HeLa	[61]
	FMDV	Block	4–6 hpi	*ND*	*ND*	PK-15	[62,63]
	EV	Transient	0–24 hpi	8 h	PKR	HeLa, RD	[64,65,66]
	PV		0–6 hpi	*ND*	*ND*	HeLa, 293T, MCF7, Vero	[67]
	CVB3		1–7 hpi	6 h	*ND*	HeLa	[68,69]
Flaviviridae	Zika	Block	24 hpi	24 h	PKR	A549, Huh7, Vero	[70,71,72]
	DENV	Unknown	6–24 hpi	12 h	PKR	A549, Huh7	[21,73]
	HCV	Oscillating	0–96 hpi	24 h	PKR	Huh7, HEK293T	[53,74,75]
Togaviridae	CHIKV	Transient	0–12 hpi	6 h	*ND*	U2OS, HEK293, Vero	[23,47]
	SFV	Transient	2–8 hpi	5 h	*ND*	MEF	[54,76,77]
	SINV	Transient		6 h	PKR	MEF	[78]
Coronaviridae	PEDV	Transient	0–36 hpi	*ND*	*ND*	Vero E6, Vero-76	[79,80]
	SARS-CoV-2	Block	0, 10, 24 hpi	*ND*	PKR	HeLa	[81,82,83]
Caliciviridae	MNV	Unknown	9, 12 hpi	9 h	PKR, GCN2	BMDM, BV2, RAW264.7	[31,84]
Arteriviridae	PRRSV	Stable	12–48 hpi	12 h	PERK	MARK-145	[85,86]
Filoviridae	EVD	Block	0.5–24 hpi	*ND*	*ND*	U2OS, Vero, Huh7	[55,87]

*ND*—Not determined. Species: Chikungunya virus (CHIKV); Coxsackie virus B (CVB3); Dengue virus (DENV); Ebola virus disease (EVD); Encephalomyocarditis virus (EMCV); Enterovirus (EV); Foot-and-mouth disease virus (FMDV); Hepatitis C virus (HCV); Murine norovirus (MNV); Porcine epidemic diarrhea virus (PEDV); Porcine reproductive and respiratory syndrome virus (PRRSV); Poliovirus (PV); severe acute respiratory syndrome coronavirus 2 (SARS-CoV-2); Semliki Forest virus (SFV); Sindbis virus (SINV); Zika virus (ZIKV), Human norovirus (HuNoV), Feline calicivirus (FCV). Duration of SG presence monitored: the time point identified in each report; also, the time point ranges differently. eIF2⍺ status: the earliest time point at which phosphorylation of eIF2α was observed or tested. Kinases: protein kinase R (PKR); protein kinase R-like ER kinase (PERK); general control nonderepressible 2 (GCN2). Cell lines: A549 (lung carcinoma epithelial cells), BMDM (bone marrow-derived macrophages), BV2 (microglial cell derived from C57/BL6 murine), HEK293T (human embryonic kidney 293 cells expressing SV40 T-antigen), HeLa (cervical cancer cells), Huh7 (human hepatoma-derived cell line), MARK-145 (monkey kidney cells), MCF7 (breast cancer cells), MEF (mouse embryonic fibroblasts), PK-15 (porcine kidney cells), RAW264.7 (monocyte/macrophage-like cells), RD (Rhabdomyosarcoma), U2OS (osteosarcoma), Vero (cells derived from kidney of an African green monkey).

**Table 2:** Role of G3BP1 and proposed mechanism of viral modulation.

Family	Species	G3BP1 Status	Role of G3BP1	Effect of Viral Protein/RNA or Titer Value		Interaction with Viral Protein	Interaction with Viral RNA	Proposed Mechanism of Action	References
				G3BP1 KD	G3BP1 OE				
Picornaviridae	EMCV	Cleavage	Anti-viral	↑	*ND*			3C protease cleaves G3BP1 at Q325	[61]
	FMDV			↑	↓	3A	Interacts with IRES	Leader protein cleaves G3BP1, G3BP1 dephosphorylated, G3BP1 binds to FMDV IRES region, 3A protein degrades G3BP1 through autophagy	[62,63,89]
	EV			↑	↓		Interacts with 3′UTR	3C proteinase cleaves G3BP1 at Q326	[64,65,66]
	PV			*ND*	↓			3C protease cleaves G3BP1, but not G3BP2, at Q326	[67,90]
	CVB3			↑	↓			3C protease cleaves G3BP1 at Q325	[69,91]
Flaviviridae	Zika	Sequestration	Pro-viral	↓	↑	Interacts with Capsid, colocalizes with envelope protein	Interacts with genomic RNA, localize with replication complexes	Sequester G3BP1 and facilitates viral replication	[70,71,72]
	DENV		Anti-viral	↑	*ND*			Subgenomic viral RNA binds to G3BP1 and antagonizes its function	[21,73]
	HCV		Pro-viral	↓	*ND*	NS5B	Localizes to vRC	G3BP1 requires at early and late stages of infection	[53,74,75]
Togaviridae	CHIKV	Sequestration	Pro-viral	↓	*ND*	nsP3	yes	Binding through FGDF motif, Reduction G3BP1 ADP-ribosylation	[23,47,92]
	SFV			↓	*ND*	nsP3	yes	Binds through FGDF motif; recruits ribosomal proteins to nsP3	[54,76,77]
	SINV			↓	*ND*	nsP3, nsP4	Colocalizes with vRNA	Binding through FGDF motif block SG assembly	[78,93]
Coronaviridae	PEDV	Cleavage	Anti-viral	↑	↓			Caspase-8-mediated G3BP1 cleavage at Asp168 and Asp169 at late infection stages	[79,80]
	SARS-CoV-2	Sequestration	Both proviral & antiviral roles have been reported	↑	*ND*	N protein, nsP1		N protein interacts/phase separates with G3BP1	[81,82,83]
Caliciviridae	MNV	Sequestration	Pro-viral	↓	*ND*	NS3, VPg	Colocalizes with vRCs	Remodels G3BP1 interactome, doesn’t affect SGs	[31,84,94]
	FCV	Cleavage	*ND*					NS6-mediated G3BP1 cleavage at E405	
Arteriviridae	PRRSV	*ND*	Not involved	No changes			G3BP1 closely associated with vRCs		[85,86]
Filoviridae	EVD	Sequestration	*ND*	*ND*		VP5		Sequestered within viral inclusions	[55,87]

*ND*—Not determined. Interaction with viral protein: identified through physical interactions. Interaction with viral RNA: identified through co-localization studies.
